# An in-depth evaluation of sample and measurement induced influences on static contact angle measurements

**DOI:** 10.1038/s41598-022-23341-3

**Published:** 2022-11-12

**Authors:** Sarah Marie Lößlein, Rolf Merz, Daniel Wyn Müller, Michael Kopnarski, Frank Mücklich

**Affiliations:** 1 Chair of Functional Materials, Department of Material Science and Engineering, Campus D3 3, 66123 Saarbrücken, Germany; 2 Institute for Surface and Thin Film Analysis, Technische Universität Kaiserslautern, Kaiserslautern, Germany

**Keywords:** Materials science, Techniques and instrumentation, Characterization and analytical techniques

## Abstract

Static contact angle measurements are one of the most popular methods to analyze the wetting behavior of materials of any kind. Although this method is readily applicable without the need of sophisticated machinery, the results obtained for the very same material may vary strongly. The sensitivity of the measurement against environmental conditions, sample preparation and measurement conduction is a main factor for inconsistent results. Since often no detailed measurement protocols exist alongside published data, contact angle values as well as elaborated wetting studies do not allow for any comparison. This paper therefore aims to discuss possible influences on static contact angle measurements and to experimentally demonstrate the extent of these effects. Sample storage conditions, cleaning procedures, droplet volume, water grade and droplet application as well as the influence of evaporation on the static contact angle are investigated in detail. Especially sample storage led to differences in the contact angle up to 60%. Depending on the wetting state, evaporation can reduce the contact angle by 30–50% within 10 min in dry atmospheres. Therefore, this paper reviews an existing approach for a climate chamber and introduces a new measuring setup based on these results. It allows for the observation of the wetting behavior for several minutes by successfully suppressing evaporation without negatively affecting the surface prior to measurement by exposure to high humidity environments.

## Introduction

Currently, literature research for “contact angle measurements” delivers more than four million publications. Hereunder, static contact angle (SCA) measurements are the most popular method to analyze a surface’s wettability - on solid metals^1,2^ or sputtered thin films^3^, polymeric surfaces^4,5^, graphene^6,7^ or even biological samples^8^. SCAs can range from hydrophilic (< 90°) to a hydrophobic behavior with a SCA above 90°. Also extreme wetting cases like superhydrophilicity with a strong droplet spreading or superhydrophobicity with SCAs around 150° can be observed by SCA measurements^9^. Their implementation is not limited to smooth surfaces, as they are also used to analyze topographically modified structures, where recently the wettability of laser treated surfaces is focused in particular^10–13^. Here, this method also allows for analyzing anisotropic wetting behavior, which is especially important for directionally patterned surfaces^11,14^. For ideally smooth surfaces, the SCA can be described by the Young’s equation and is considered as a thermodynamic contact angle reached by energy minimization in the three-phase wetting system consisting of liquid, solid and vapor^9,15^. Real surfaces are characterized by roughness either fully wetted in the Wenzel wetting state^16^ or by partial wetting with air inclusions between the droplet and topographic features (Cassie–Baxter state^17^), resulting in a chemically heterogeneous surface.

Already in the 1980’s researchers were aware of the strong scattering of contact angles on metallic surfaces like copper due to sample contamination, cleaning agents and cleaning procedure^18^. More recent studies could proof that especially a contamination with hydrocarbons plays a major role in the wetting behavior of solid materials^19^, as their adsorption can increase SCAs on flat copper samples from 45° to 100°^2^. Cleaning agents can influence the composition of this airborne contamination layer and therewith alter the SCA results^19^. In 2015 Long et al.^20^ showed that the adsorption layer also is highly dependent on sample storage conditions. Apparently, different studies have shown reasons for the broad scattering of contact angles in literature for the same material and various factors independent of the sample surface as droplet volume, temperature or humidity are well known^21^. However, no fixed measurement protocols exist including all possible sample or measurement induced influences and focusing on the dependence between sample contamination and resulting contact angle, which seems to be a key factor in any wetting analysis. For the dynamic measurement of receding and advancing contact angles Huhtamäki et al.^22^ as well as Drelich^23^ provide a protocol of the measurement steps by gradually increasing and afterwards decreasing droplet volume including indications of potential measurement influences. They claim that the SCA measurement using the sessile drop method might only depict metastable results as the drop could be in any local instead of the global minimum. But different studies showed, that also dynamic contact angle measurements are often subjected to droplet disturbing effects, e.g. baseline pinning or vibrations transferred from the needle to the droplet in the sessile droplet setup^24^ keeping the droplet from reaching the global thermodynamic minimum. Often natural vibrations in the laboratory prevent the contact angle from reaching theoretical values^25^. Moreover, there is a lack of theoretical understanding of the resulting contact angle hysteresis^24^ while there are well defined models for SCA measurements based on the Young’s equation for ideal surfaces and on the Wenzel- and Cassie–Baxter models for real surfaces depicting roughness^15–17^.

On top of that, especially the measurement of the receding contact angle poses a challenge if the wetting behavior is yet unknown. The starting volume needs to be estimated and can reach values up to 150 µl^22^ resulting in excessively large droplet diameters strongly limiting the number of measurement replicates per sample.

In contrast, SCAs are attractive due to the simplicity of the method and so they are the established values in literature to describe the wettability of a surface, which is why they cannot be substituted by other approaches but rather supplemented to improve data interpretation^26^. For SCA measurements a detailed discussion about potential sample or measurement induced influences also showing the extent of their impact on hydrophilic as well as hydrophobic surfaces could not be found in literature.

**Figure 1:**
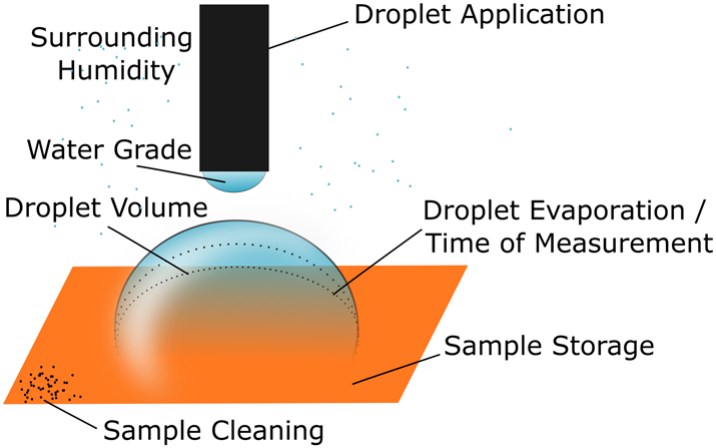
Overview of influences on static contact angle measurements covered in this paper.

Because of the popularity of the SCA measurement, its simplicity and broad applicability on different materials and sample sizes, this paper aims to provide a detailed analysis of different measurement parameters and how an uncontrolled alteration of the results can be avoided. Sample induced influences as sample preparation, storage and cleaning are investigated and compared to measurement induced influences such as droplet volume, application method and many more (compare Fig. 1). Time of measurement and its connection to droplet evaporation is discussed in detail with a review of a climate chamber as suggested by Drelich^23^. A new method that allows for an observation of the droplet throughout several minutes by successfully suppressing evaporation without distorting the contact angle results is presented. Based on the provided analyses, a basic framework for SCA measurements can be set allowing for a better comparability of different wettability studies in the future.

## Materials and methods

Copper was chosen as a model material due to its broad application in research and industry^13,27–29^ and its common use in wetting analyses as discussed earlier^2,18,20,30^. Copper coupons (10 × 25 mm^2^) were mirror polished to an averaged roughness of 3.3 ± 0.6 nm measured by confocal laser scanning microscopy (*Olympus LEXT OLS4100*), where the preparation routine was published in an earlier work^31^. After preparation, the samples were cleaned in ethanol in an ultrasonic bath for three minutes and dried with an ambient air stream. Right before contact angle measurement, dust and particles were removed via an ambient air stream from a fan. Pressured air was avoided due to possible oil contamination^22^. Wetting tests were performed on a *Krüss DSA 100* equipped with a 100 µl Hamilton syringe at room temperature (22 °C/~ 20% relative humidity). If not mentioned otherwise, measurements were taken 5 s after droplet application with a droplet volume of 3 µl. Images of the experimental setup for sessile drop measurements as conducted in this work together with an image of a droplet applied on a copper substrate can be found in the Supplementary Material (Supplementary Fig. S1a). The 3 µl droplet is dosed automatically with 2.5 µl/s. A fully automated program was written in the *Advance software Version 1.13* defining positions for droplet dosing and application to allow for comparable measurement conditions for all experiments keeping the influence of experimental errors as small as possible. Five seconds after droplet application a high-resolution camera takes three images of the droplet within 1 s. The *Advance software* allows for a fully automated fit of the droplet and calculation of the resulting contact angle on both sides of the droplet as well as the volume using the elliptical fit method of the software. According to the software manual the ellipse fit method is recommended for a contact angle range between 10° and 120° and therefore applicable for the analyses conducted. The fit method adapts the optically detected contour of the droplet to a conic section equation. Tangents are placed through the three-phase point. The contact angle is calculated from the average angles on both sides of the droplet^32^. This was performed on three images per droplet and results were averaged to determine the SCA of the droplet (labelled as “SCA” in figures). It should be noted that in this study the measurement of copper surface wettability is primarily used to monitor the effect of the different conditions. Therefore, results are normalized to solely show the effect under investigation. Data normalization was based on a reference sample state that was defined for each experiment. The individual SCAs measured on the reference state were averaged to *Mean SCA_Reference*. Whenever possible all parameter variations were carried out on the same sample as the reference measurements, but in different sample areas. All experiments were conducted on multiple samples to ensure sufficient statistics. If not mentioned otherwise, normalization as shown in Eq. (1) was carried out sample wise to equal out differences resulting from other sources than the parameter under investigation. A detailed example of normalization calculations is given in the supplementary material (compare Supplementary Table S1).

1 $$Normalized \, SCA= \frac{SCA/^\circ }{Mean\, SC{A}_{Reference}/^\circ }$$

The reference sample is specified for each experiment. Results are displayed in box plots calculated by *OriginPro 2019* with half boxes on the left and randomly scattered data points on the right. For each experiment, a new set of samples was used to make sure that no sample area came in contact with water twice and that no experimental parameters under investigation overlap. All experiments were conducted on initially hydrophilic and hydrophobic samples adjusted by different storage conditions. Sample storage conditions were defined based on the experimental results in the chapter “Sample Storage”. The sample preparation and storage as well as pretreatments specified in this section were kept identical for all experiments.

The analysis of the surface chemistry was done by X-ray photoelectron spectroscopy (XPS) with an *Axis Nova surface analysis spectrometer* (*Kratos Analytical Ltd.*). In this system, photoelectrons are released from the sample’s surface by monochromatic Al Kα radiation of 1486.6 eV at a working pressure of 10^−8^ mbar and analyzed according to their kinetic energy by an electrostatic hemispherical sector analyzer. Relative atomic percentages were calculated by the intensities of elemental photoelectron lines, measured in survey spectra at a high electron analyzer pass energy of 160 eV and standard sensitivity factors supplied by the spectrometer manufacturer. Contributing elemental binding states were identified using detail spectra, measured with a lowered pass energy of 20 eV and therefore 8 times elevated energy resolution. Here, each binding state of an element shows a significant slight chemical shift in comparison to its elemental species. The given ratios of different C, O and Cu binding states is based on a deconvolution assuming a Shirely type background for the inelastic scattered electrons and Gaussian/Lorentzian line-shapes for each contributing binding state. The XPS information volume is given by the lateral measuring zone (0.35 mm × 0.70 mm) times the maximum escape depth of the photoelectrons, which is typically about 3–5 nm, depending on their kinetic energy and the material they have to pass.

Static Time-of-Flight Secondary Ion Mass Spectrometry (ToF–SIMS) measurements for surface spectroscopy and imaging were performed using an *ION-ToF IV instrument* (*ION-TOF GmbH, Münster, Germany*). The primary ion gun was operated with Bi3+ ions with an energy of 25 keV and the emitted secondary ions were detected in positive and negative polarity.

## Results and discussion

### Sample induced influences

#### Sample preparation

Recently many studies aim at tailoring the wetting response by applying periodic patterns with pulsed lasers^33,34^. An earlier study proved the strong influence of surface chemistry on the wetting behavior of topographically altered samples^11^, which is especially important for laser patterned surfaces as the modification of topography always is accompanied by distinct changes in surface chemistry through oxidation processes^35^. Researchers are well aware of the influence of topographical changes e.g. by laser patterning^10,33,36–40^. Nevertheless, so far no generally valid statements can be made about the influence of topography on the wetting behavior, especially as influences of topography and chemistry are often interconnected and metastable wetting states or transitions from Wenzel to Cassie–Baxter wetting can be observed^41^. To make sure that such an influence does not overlay the effect under investigation in this work, flat samples were chosen as a substrate material. The sample preparation usually is directly connected to the surface topography, that proved to have a great influence on the wettability of a surface^42^. If the wettability of a solid material is to be investigated, the influence of topography should therefore be kept as small as possible by following effective preparation routines. A R_a_-value below 500 nm is a reference value to avoid topographical influence on the SCA^21^. With the routine presented in Ref.^31^ and applied on the samples, the average roughness R_a_ of a cold rolled copper sheet could be reduced to 3.3 ± 0.6 nm. Though the surface is close to an ideal surface with the small roughness, in reality also such a minor topography contradicts the theoretical wetting according to the Young´s equation and rather supports a uniform Wenzel wetting of the surface without droplet pinning.

#### Sample storage

**Figure 2:**
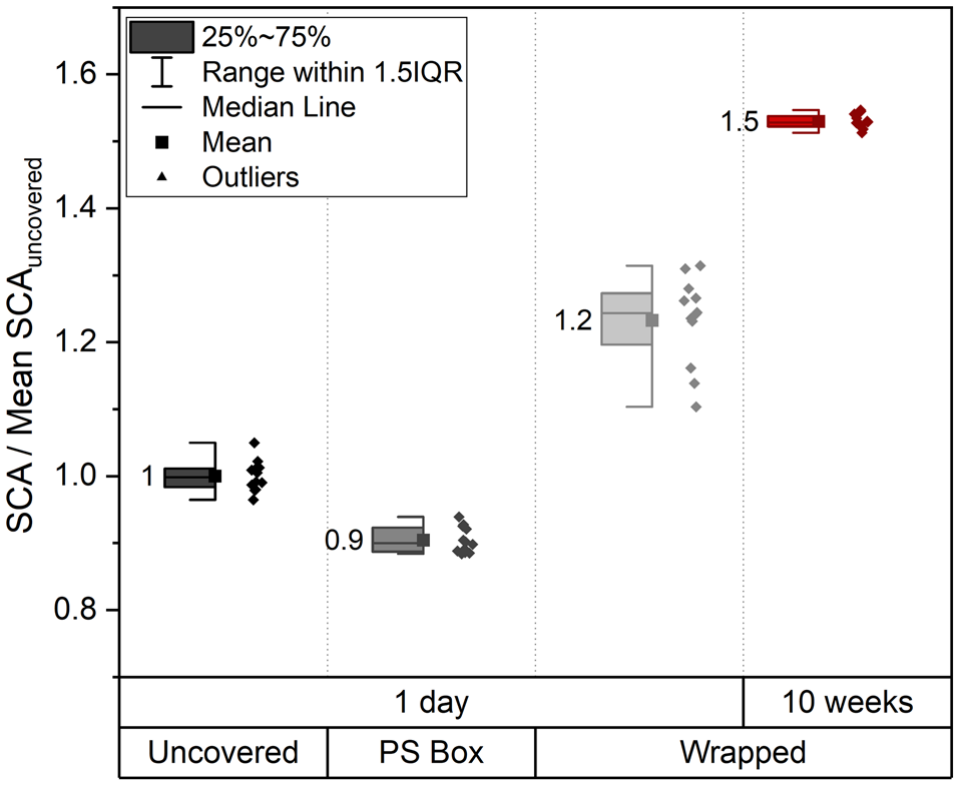
Contact angle of a 3 µl droplet on polished copper samples stored without any cover (uncovered), in a common polystyrene sample box (PS Box) or wrapped in a woodfree tissue (Wrapped). For the wrapped samples a storage time of one day is compared to 10 weeks of storage. The different sample packaging methods lead to different contact angles although all were stored under the same ambient atmospheric conditions. Per storage condition two samples were investigated by applying 6 droplets each. The reference data for normalization is “uncovered/1 day”. Here data normalization was not done sample wise but with the total mean value of the two samples stored for one day uncovered as separate samples had to be used for every storage condition.

While sample preparation with its resulting topography and chemistry are broadly discussed in the wetting community, little attention is paid to the storage conditions after sample preparation. As the wetting response especially of topographically altered surfaces is highly dependent on adsorbed carbon groups^20,43,44^, storage plays a major role in the development of the wettability behavior. Different gaseous environments lead to differences in the aging related contact angle development^20^. Usually, atmospheric conditions are chosen as the atmospheric carbon leads to the time dependent hydrophobization of metallic surfaces by adsorption of aliphatic carbon groups^20,27^. As the composition of the surrounding atmosphere already proved to have a great influence on contact angles, we investigated, if different storage conditions in the same atmosphere also alter the contact angle. Samples were prepared in one batch, cleaned via immersion in ethanol in an ultrasonic bath and stored either uncovered and unboxed, uncovered in a conventional sample box (polystyrene) or wrapped in a woodfree tissue (laservision A99CLSTA1302) in an identical box. The different protected samples are stored in the same atmospheric environment for one day (≈ 22 h). In Fig. 2 they are compared to identical copper samples stored in the wrapping paper for ten weeks. Due to the prolonged storage time the samples were packed in air tight boxes with silica gel beads to avoid corrosive effects.

Although the samples were polished, cleaned and stored for 1 day in one batch under the same atmospheric conditions, the different additional protection measures lead to contact angle differences up to 30%. After 10 weeks in the woodfree tissue the SCA is increased even more by 50% compared to the reference sample. This experiment clearly demonstrates that also flat samples are subjected to aging induced wettability conversions, which are most likely caused by different carbon adsorption rates depending on the amount of carbon provided by the surrounding materials. We assume that the observations made by Long et al.^20^ regarding the hydrophobization of laser patterned copper surfaces by adsorption of aliphatic carbon groups are also applicable to copper surfaces undergoing preparation without involvement of laser treatment. More recent studies underpin this assumption^2^, and suggest that not only different atmospheres, but also different cleaning procedures lead to the incorporation of certain carbon species in the adsorbate layer^19^ that strongly affect the resulting wetting behavior.

For sample storage the results displayed in Fig. 2 suggest that different protection measures lead to altered micro-environments for the individual samples: The uncovered sample is exposed to a natural aging environment, while for the samples in a polystyrene box the carbon supply is restricted to the air volume inside the box. Less volatile carbon is available that can adhere on the sample, which is why they turn out more hydrophilic than the uncovered reference samples. On the other hand, the woodfree tissue seems to act as a carbon-donor increasing the amount of free carbon groups close to the sample surface and supporting their adsorption resulting in an increase of the SCA by 20% during one night of storage. Prolonged storage times seem to further increase the carbon adsorption as well as the resulting hydrophobicity.

To gain a deeper understanding of the influence of the wrapping paper, the paper itself as well as samples tightly wrapped in the paper for ten weeks (compare red data in Fig. 2) were analyzed by means of XPS-measurements. The SCAs of three samples were averaged to 108° ± 1°. Table 1 summarizes the results of the overview spectra and the C 1s detail spectrum for three measurement spots on a wrapped sample and the wrapping paper. The C1s region contains signals from carbon-species in three different binding states: carbon bound in aliphatic and aromatic hydrocarbon at an electron binding energy of 285 eV, carbon with one oxygen as binding partner at 286.6 eV (typical for organic hydroxyl or carbonyl groups), and carbon with two oxygen atoms as binding partners at 288.4 eV (typical for carboxyl groups). In the following, carbon bound in nonpolar aliphatic or aromatic hydrocarbons (signal at 285 eV) is called “nonpolar carbon”, while the sum of the two others with one or two oxygen atoms as binding partners is called “polar carbon”. The oxygen region of the spectrum contains always at least two signals: they stem from oxygen bound in hydroxyl and carbonyl groups at 531.2–532.3 eV and oxygen bound in carboxyl groups at 532.2–533.6 eV^45^. Only the sum of the signals in the oxygen region is used here as the deconvolution of the peaks is subject to large uncertainties. Oxygen bound as water would appear also at 533.2 eV, so the presence of adsorbed water films can’t be excluded.

**Table 1:** Averaged XPS-results of the copper surfaces wrapped in the woodfree tissue and of the wrapping paper itself.

	C [at.-%]	Cu [at.-%]	N [at.-%]	O [at.-%]	S [at.-%]	C–C/C–H [at.-%]	C–O [at.-%]	O–C–O [at.-%]
						Nonpolar carbon	Polar carbon	
Copper sample	53.79 ± 0.19	22.64 ± 0.45	0.67 ± 0.08	22.46 ± 0.16	0.44 ± 0.11	44.78 ± 0.13	4.74 ± 0.04	4.27 ± 0.08
Wrapping paper	61.69 ± 0.06		0.36 ± 0.01	37.86 ± 0.09	0.08 ± 0.09	11.69 ± 0.12	39.49 ± 0.11	10.51 ± 0.05

As expected, the copper samples show a strong carbon signal as well as an oxygen signal due to native oxidation^46^. Minor nitrogen and sulfur contamination could be either transmitted by the wrapping paper or be a result of the sample preparation. The detailed C 1s-spectrum reveals a dominance of nonpolar carbon being responsible for the hydrophobic wetting behavior^20^. Interestingly, the paper itself shows a more pronounced polar carbon–oxygen content that does not seem to be transferred to the sample surface. A possible reason could be the strong bond of C–O in the cellulose fiber. The aliphatic carbon, in contrast, might only be adsorbed on the paper surface and can therefore be donated to the copper sample with its reactive surface after sample preparation. Therefore, the results in Table 1 suggest that cellulose-based paper can adsorb aliphatic carbon groups and act as a donor for these functional groups that leave the sample surface in a hydrophobic state. The small standard deviation for the nonpolar carbon on the copper samples indicates that for a tight wrapping a homogeneous transfer of the carbon groups on the sample surfaces is possible. TOF–SIMS analysis was performed to gain a better understanding of the carbon exchange between the wrapping paper and the sample.

**Figure 3:**
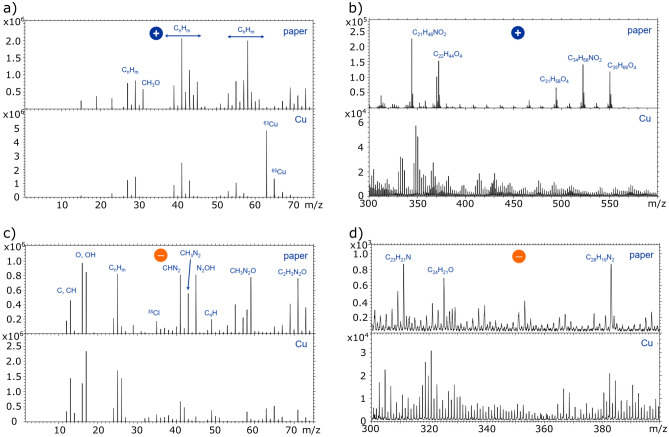
Positive (**a,b**) and negative (**c,d**) secondary ion spectra received on the stored sample (‘Cu’) and on the paper (‘paper’) sample are shown in a region of low masses (< 75 amu) and in a region of higher masses (> 300 region). The low mass secondary ion spectra of the Cu sample indicate a rather clean metallic sample with slight contaminations of typical environmental simple hydrocarbons. Some corresponding peaks are also found on the paper, but the observed peak groups differ. In the region of higher masses (> 300 amu), which is significant for more complex organic molecules, the dominant peaks found on the paper at 311, 325 and 383 amu were not found on the Cu sample, which indicates that there is no complex component of the paper material like cellulose molecules transferred to the sample.

**Figure 4:**
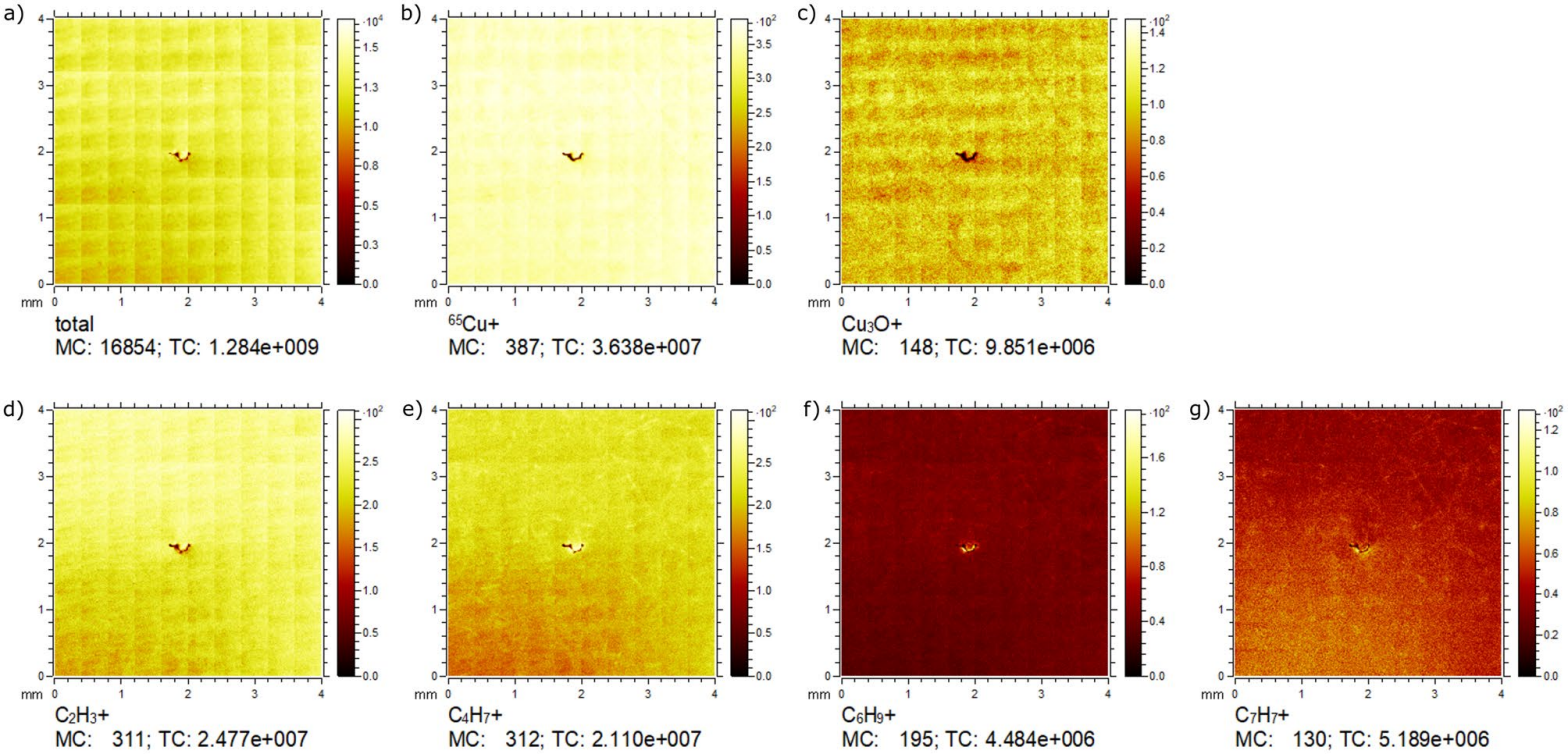
Lateral distribution maps of selected positive secondary ion intensities as macro raster 4 × 4 mm^2^ show a homogeneous allocation: (**a**) total positive secondary ions, (**b**) ^65^Cu^+^, (**c**) Cu_3_O^+^, (**d**) C_2_H_3_^+^, (**e**) C_4_H_7_^+^, (**f**) C_6_H_9_^+^ and (**g**) C_7_H_7_^+^. An accidental particle contaminant was used for adjustment and the grid structure is an artefact of measurement in stitching mode.

The resulting spectra of positive and negative secondary ions with lower masses (< 75 amu) in Fig. 3 indicate that there is a usual contamination of simple nonpolar hydrocarbon molecules on the Cu surface after storage and contact to the paper, which can be interpreted as caused by environmental hydrocarbon adsorptions. On the paper this contaminant may be present but overwhelmed by other small organic components. In the higher mass region, significant for more complex molecules (> 300 amu), the dominant and characteristic peaks, which were found on the paper (311, 325 and 383 amu), find no correspondence on the sample surface. Side experiments, where the paper was manually pressed or even rubbed on the surface, also did not show corresponding peak groups. These measurements therefore support the thesis of the transmission of adventitious carbon initially adsorbed on the paper to the sample instead of actual paper components being transferred. The very small standard deviation of the corresponding SCA of only 1° (0.9%) indicates an even distribution of the adsorbed carbon on the sample surface. The homogeneous lateral intensity distribution of positively charged secondary ions as shown in Fig. 4 shows that the tight wrapping of the samples leads to an even transfer of carbon groups on the wrapped surfaces and therewith guarantees a stable wetting behavior in different sample areas.

Based on these results, it is possible to create both hydrophilic and hydrophobic surfaces of the same substrate simply by the control of the storage conditions. The different influences on SCA measurements investigated in this work were explored on both hydrophilic and hydrophobic surfaces. To create hydrophilic copper surfaces after preparation, one half of the sample batch was stored for one day in a polystyrene box, resulting in an average SCA of 62° ± 3° under standard measurement conditions used as reference conditions in the following experiments. To create hydrophobic surfaces, the results presented in Fig. 2 were applied and the other half of the polished copper samples were wrapped in the woodfree tissue, placed in individual single sample boxes which are then stored in airtight boxes filled with silica gel beads to keep the humidity low in order to create a strong and homogeneous hydrophobicity. With an averaged SCA of 106° ± 1° after 2 weeks of storage under standard measurement conditions, the desired hydrophobicity of the surfaces was achieved.

#### Sample cleaning

As discussed before, it is well known and accepted in literature that carbon containing adsorption layers strongly stimulate the wetting behavior of metallic substrates as the carbon adsorption reduces the surface energy^19,20,27,47^. Still, some researchers argue that organic matter would only play a minor role in the hydrophobization of metallic surfaces^48^ and rather attribute the hydrophobization process to reactions of the surface with oxygen^33,48,49^. If oxidation was the main factor responsible, sample cleaning with solvents should not influence the contact angle significantly.

**Figure 5:**
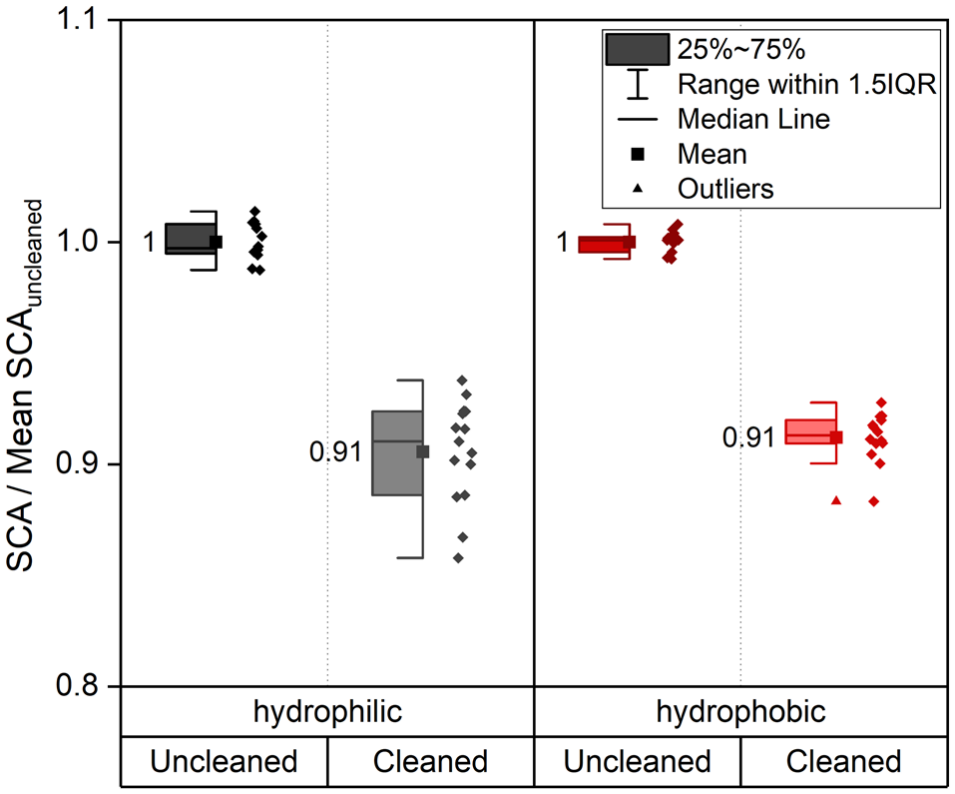
Influence on the SCA of ultrasonic cleaning in ethanol of hydrophilic and hydrophobic samples. For both wetting types SCAs before the cleaning (uncleaned) were compared to the SCA after cleaning (cleaned). Fifteen droplets were applied on three samples per condition. The reference data for normalization is “uncleaned”. For both wetting types a separate normalization was carried out.

Figure 5 shows, how a 5-min ultrasonic bath in ethanol strongly lowers the contact angle of hydrophilic as well as hydrophobic samples. Before the cleaning, the SCA was measured on different areas on one half of the sample, the other half was measured after the respective ultrasonic cleaning. All results are standardized to the mean value of the measurements before the cleaning for every sample (uncleaned).

Interestingly, both samples show the same decrease in contact angle through the cleaning in ethanol of 9%. Heier et al.^19^ showed that cleaning agents on the one hand remove some of the carbon groups and incorporate functional groups into the adsorbate layer on the other hand. Due to the mixed polarity of ethanol it is believed to remove the nonpolar as well as polar sites on the sample surface leaving it in a more hydrophilic state - as clean copper is hydrophilic^2^. The reduction of the contact angle by solvent cleaning is in good agreement with results from 1985^18^, where a contact angle dependency of the cleaning solvent and cleaning duration was observed. The results shown in Fig. 5 indicate that the contamination layer on the sample surface indeed plays a major role in the development of hydrophobicity. The above experiment shows that while a simple cleaning in alcoholic solvents is considered as a good practice before sample imaging or analyses, for SCA measurements it has a significant influence on the measured angles. This means that no general recommendation for an “appropriate cleaning” before SCA measurements^23^ should be made as often especially the composition of the adsorbed carbon layer is of great interest and might be easily disturbed by a preceding cleaning. So, if researchers decide to perform a cleaning before the measurement, they should be aware of the strong effect and perform the same cleaning for all samples to assure comparable conditions. Even hydrophilic samples only stored for one day pile up enough carbon to show a reduction of the contact angle after the ultrasonic cleaning.

Evidently, for reproducible SCA measurements many details need to be considered before the actual measurement can be conducted. Though wetting behavior of different materials has been studied extensively in the past, there is still a lack of knowledge of basic relationships between sample properties, aging behavior, and the resulting contact angles. Especially for systematic studies investigating the influence of, e.g. the microstructure, strain, micro-topography but also of surface chemistry it is of the utmost importance to create comparable sample conditions in regards of preparation, storage and sample cleaning.

Even though the general protocol of a SCA measurement seems simple and not error-prone, no standard values for the volume of the dosed liquid or the exact measurement time or the droplet application method exist. Therefore, the next section of this paper aims at investigating the influence of different parameters in the measurement protocol and setting up a universal recommendation for SCA measurements. Especially the time of the contact angle determination will be discussed to highlight the competing effects of evaporation on the one hand and the required time to reach a stable wetting condition on the other hand.

### Measurement induced influences

#### Probing liquid

**Figure 6:**
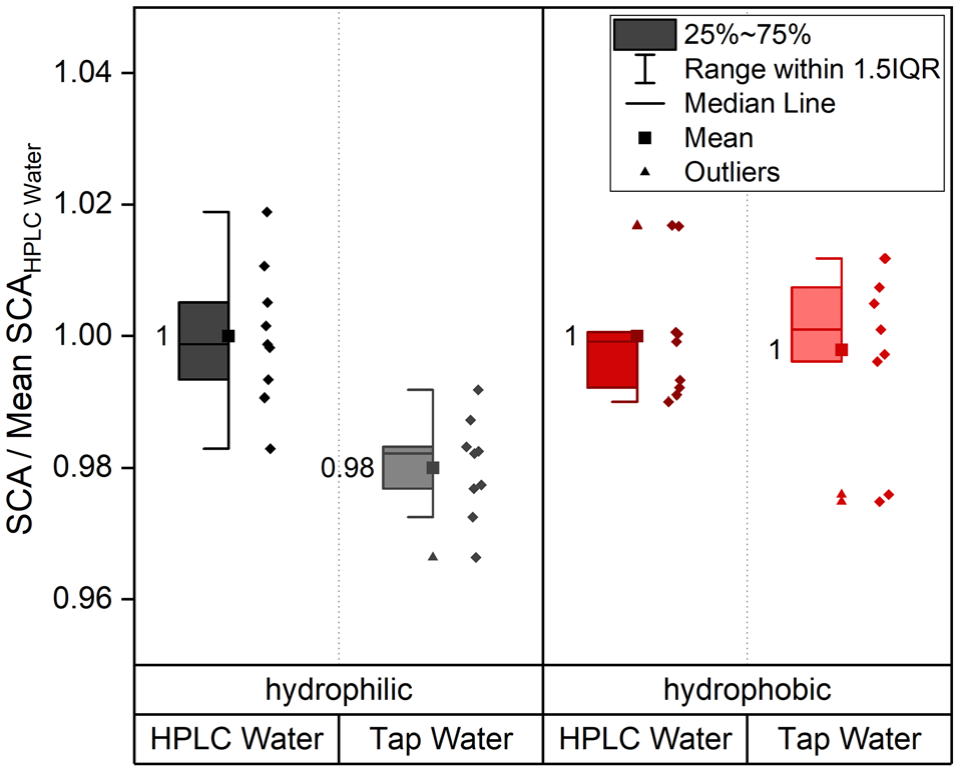
SCA measurements with HPLC grade water (0.91 µS/cm) compared to tap water (> 300 µS/cm). Measurements were performed on three different samples with three droplets per sample per water grade and sample condition. The reference data for normalization is “HPLC Water”. For both wetting types a separate normalization was carried out.

Similar to sample conditioning, the probing liquid used to measure the SCA should be chosen carefully. ASTM set standards for laboratory water by classifying it into four types^50^. Electrical conductivity is next to the pH-value and other specifications like the total organic carbon the main criteria for water classification. The four ASTM types range from an electrical conductivity of 0.056 µS/cm (type I) to 5 µS/cm (type IV). Conductivity is increased by the amount of free ions in the water where a low conductivity is favorable for chemical analyses (usually type 2 or lower (conductivity < 1 µs/cm, 25 °C))^51^. So far, the sensitivity of SCA measurements towards the water quality used has not been discussed until now in the literature, to the best of our knowledge. Therefore, two significantly different qualities of water were compared as shown in Fig. 6. On the same samples, contact angles were measured using HPLC gradient grade analyses water with a conductivity of 0.91 µS/cm as well as standard tap water with a conductivity of 309 µS/cm, according to the annual local water analyses report (2020). Measurements were conducted with a pipette to avoid a contamination of the microliter syringe with the tap water.

As shown in Fig. 6, no significant differences were observed for the hydrophobic samples while the tap water formed lower contact angles on the hydrophilic samples than the purified HPLC grade water. We believe that the thicker carbon rich contamination layer on the hydrophobic samples (compare section “sample storage”) shields the copper surface from the droplet and reduces the interaction forces between the substrate and the water. Noble materials like copper or gold interact via long-ranged van der Waals forces with the liquid^7,52^ that might be active for the hydrophilic samples, but interrupted by the more pronounced carbon layer for the hydrophobic samples. So, while we assume that on the hydrophobic samples the water forms interaction bonds with the carbon contamination layer, on the hydrophilic samples it rather might interact with the copper substrate leading to a different behavior of the two sample types.

Another possible factor is the altered dissolving properties of the tap water due to a higher ion concentration, and a resulting solution of the adsorbed carbon layer in the water with increased wettability of the surface.

Moreover, the tap water can be rather qualified as a molecular colloid than a pure liquid, which might also influence the surface tension of the water and therewith its wetting properties. The higher salt content of the tap water might also cause corrosion of metallic surfaces that are less capable of forming passivation layers than copper^46^. Especially long-time wetting tests, where droplets might be observed for several minutes might be prone to such corrosive reactions, why we recommend opting for purified water whenever possible.

Also numerical simulations of the wetting behavior of surfaces as presented in Refs.^2,53^ usually are based on the assumption of molecular pure water. Therefore, it is seen as a good practice to use purified water for the experimental data the simulations are built on.

#### Droplet volume

Especially the volume of the deposited droplet is vastly varied in different studies. Volumes of water droplets range from 2 to 3 µl^12,54–57^ to significant higher values from 7 to even 10 µl^34,58–60^. Sometimes, only vague information on the droplet volume or volume ranges are provided^37^. In theory, the contact angle of ideal surfaces should not be influenced by the droplet volume. Seo et al.^61^ proved that superhydrophobic surfaces can fulfill the requirements for this volume independence of the SCA. For superhydrophobic samples droplet deposition can pose a challenge, especially for surfaces with low water adhesion. An increase in droplet volume can be useful here to allow the droplet to fall from the needle^62^.

**Figure 7:**
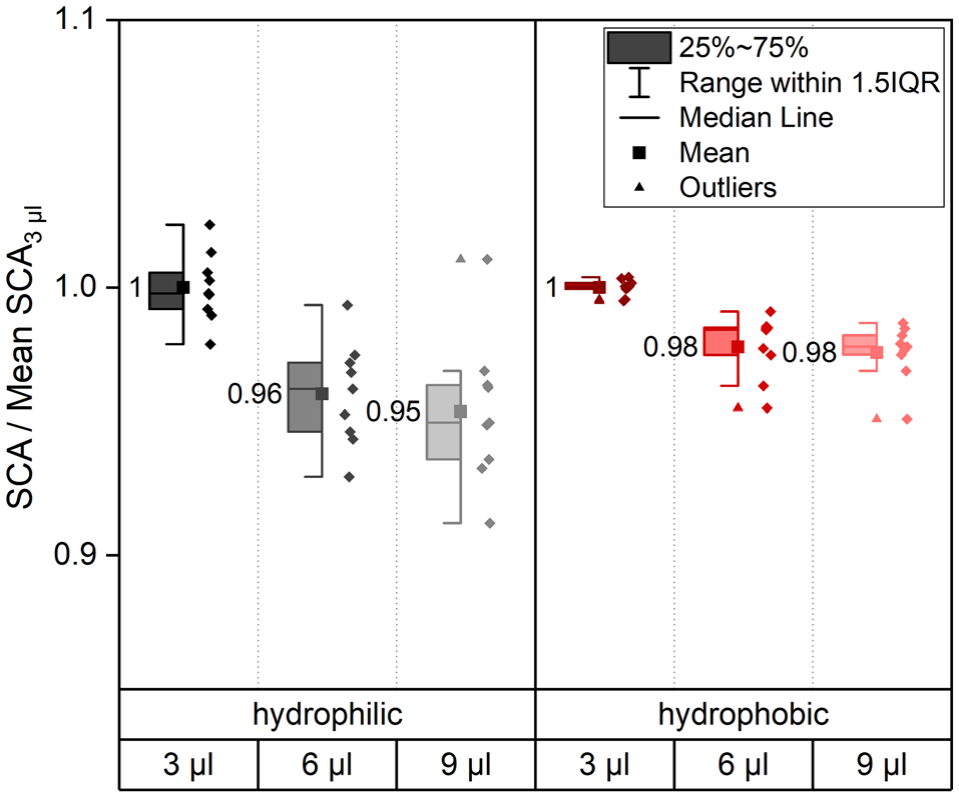
SCA measurements with different droplet volumes. For direct comparison of the influence of the droplet volume contact angles on the same sample were measured with 3 µl, 6 µl and 9 µl droplets. In total three samples were analyzed per wetting condition with three droplets of each volume per sample. The reference data for normalization is “3 µl”. For both wetting types a separate normalization was carried out.

To investigate the effect of droplet volume on hydrophilic and hydrophobic, but not superhydrophobic samples, droplets of three different volumes (3, 6 and 9 µl) were applied (compare Fig. 7).

The influence of higher droplet volumes on more hydrophilic samples can be traced back to the impact of gravity that is increasing with the droplet volume. Due to the improved surface wetting, the SCA decreases more for hydrophilic samples than for hydrophobic samples. Results in Fig. 7 indicate that smaller droplets deliver more accurate results with less deviation and fewer outliers. Nevertheless, droplets should also not be too small to avoid excessive disturbance through the needle during droplet application^22,23^. For very small droplets with a diameter in the µm range, line tension must be considered as it may vary with droplet diameter^63^. For the volumes applied here with diameters in the mm range, gravity is the dominating factor. The cosine of the SCA and the reciprocal of the droplet radius show a linear relation for both wetting types (see Supporting Fig. S2) suggesting an independence of the line tension from the applied droplet volumes^63^. A volume of 3 µl appears to be ideal to minimize the influence of gravity^12,64^ and to produce stable droplets that are not disturbed too easily by vibrations at the same time, while the droplet diameter is still small enough to allow for multiple measurements on an averaged sample size. Here, it must be highlighted that the considerations above are based on SCA measurements on topographically flat and chemically homogeneous samples. For heterogeneous sample surfaces, the droplet volume must be sufficiently high to average the surface heterogeneity. Marmur et al.^25^ recommend a factor of 10^3^ between the scale of heterogeneity and droplet size. This recommendation might not be applicable to all sample-water-systems e.g. a laser line pattern with a periodicity of 50 µm as analyzed in^11^. Especially if anisotropic wetting behavior is to be investigated^11,65–68^ excessively large droplets might simply flood the entire sample and cover the effect of anisotropic structures. Therefore, we recommend using the smallest possible droplet volume/diameter to achieve reproducible results on different areas of the heterogeneous sample.

#### Droplet application

**Figure 8:**
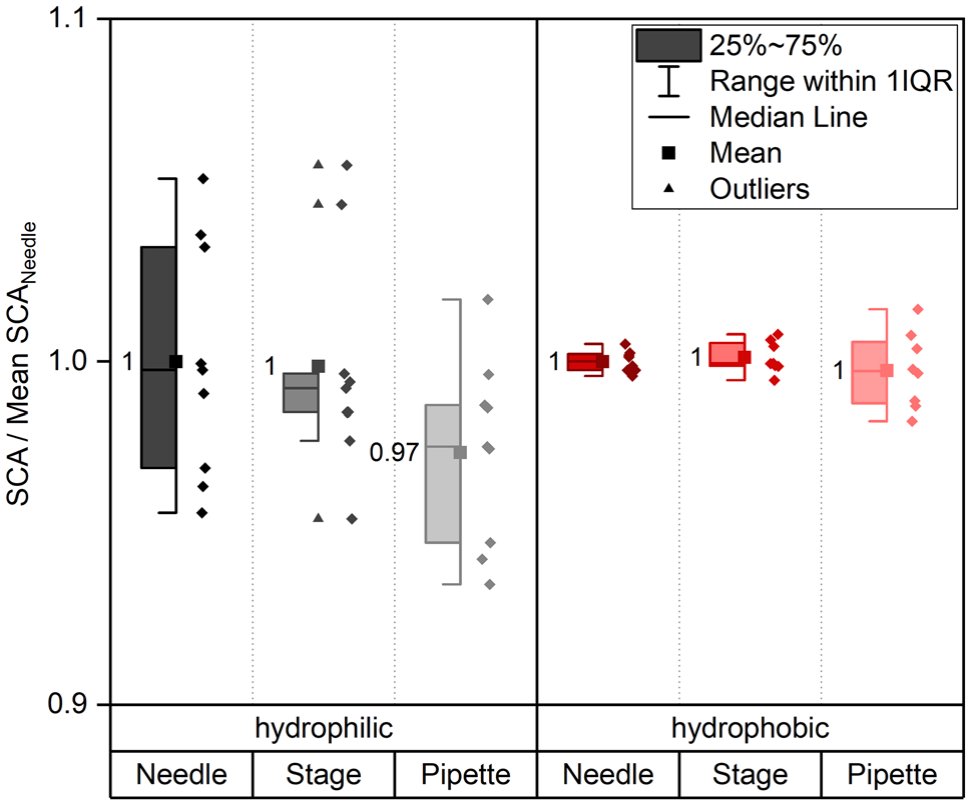
Comparison of different kinds of application of a 3 µl water droplet. On the same sample droplets were applied with the three methods, i.e., by moving the needle (Needle), by moving the stage (Stage) or by manual pipetting (Pipette). Measurements were repeated at least 8 times for each method on three samples. The reference data for normalization is “Needle”. For both wetting types a separate normalization was carried out.

Once the droplet volume is set, different options for droplet application during SCA measurements are available: this can be done by dosing the droplet, leave it hanging free from the needle tip and lifting the stage until the droplet can be picked up by the sample. This procedure of droplet application (“Stage”) contrasts with the current state of the art with increasing automation of contact angle measurements where usually the needle positions are defined (“Needle”), and the stage height is kept constant. While standard devices are equipped with a microliter syringe and a needle to dose and apply the droplet, in self-built devices, pipettes (“Pipette”) can be an option to dose the exact volume. Figure 8 shows the comparison of the three different application methods. At least eight 3 µl-droplets were applied on three samples per wetting condition and application method. The reference sample for data normalization was the “Needle”-application. The results are displayed in Fig. 8 for hydrophilic and hydrophobic samples. On the hydrophilic samples, pipetting of the droplet leads to an average reduction of the SCA of 3%, whereas no deviation can be observed on the hydrophobic samples between the different application methods. We believe that the increased hydrophobicity and the associated higher energy barrier of the samples results in a decreased pressure sensitivity during droplet application. On the hydrophilic samples, in contrast, the enhanced pressure during the manual pipetting of the droplet compared to the defined gentle application by needle or stage leads to a stronger spreading of the droplet. Therefore, a reduction of the SCA is observed. On the hydrophilic samples all application methods lead to a stronger scattering of the contact angle, which might be the result of a more inhomogeneous carbon agglomeration of the samples after one day of storage (compare section “Sample Storage”). In contrast, on the hydrophobic samples only the droplet application with the pipette results in a stronger scattering of contact angles, which might be due to a less reproducible application force applied by the researcher. In general, the needle or stage method is recommended. Still, the experiment shown in Fig. 8 proofs that for extreme wetting cases (needle tip more hydrophilic than the surface under investigation) or in case of a lack of automated equipment, gentle droplet application with a pipette can be a reasonable alternative. For hydrophilic samples a pipette application is not recommendable.

#### Humidity

Usually, humidity and temperature can be controlled, e.g. by air conditioning. Additionally, silica gel can be used to lower the humidity and avoid corrosion of the samples during storage. Still, slight changes in both parameters often cannot be avoided for example due to sample transportation. While common laboratory air conditioning can control the temperature easily, humidity control poses a challenge as conventional air conditioners do not allow for a hygrostat-controlled humidity regulation. Moreover, slight changes in temperature are not expected to influence the resulting contact angle as fluctuations between 20 and 40 °C do not alter the surface tension of water significantly^21,22,69^.

**Figure 9:**
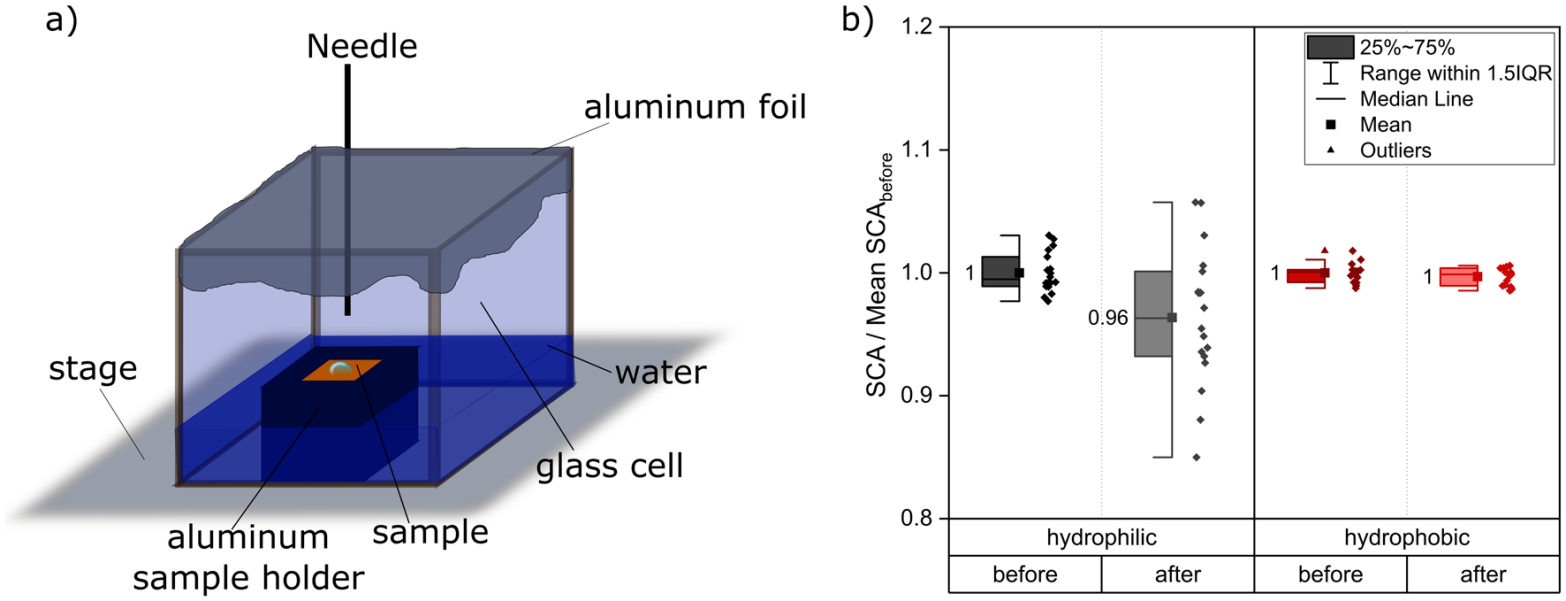
(**a**) Schematic of an experimental setup to avoid droplet evaporation as suggested by Drelich^23^. (**b**) Contact angle of hydrophilic and hydrophobic samples before and after 15 min of exposure in setup (**a**). The experiments were repeated on three samples for the hydrophobic wetting condition with five droplets each and on four samples for the hydrophilic wetting condition as some samples could only fit four droplets due to the enhanced spreading. The reference data for normalization is “before”. For both wetting types a separate normalization was carried out.

Humidity plays a major role in contact angle measurements when it comes to the reduction of droplet evaporation during long term measurements. To avoid evaporation, the atmosphere around the samples usually is saturated with water. Commercially available climate chambers additionally offer the possibility of changing the atmosphere to inert gases like nitrogen. Attention should be paid here because also alleged inert gases like nitrogen might be adsorbed on the sample and influence the contamination layer before the measurement. Drelich^23^ showed a simple setup to suppress evaporation by putting the sample under investigation in a glass cell filled with the probe liquid and covered with parafilm. After a few minutes a saturated atmosphere should be generated inside the cell and droplets can be applied by the needle penetrating the cover of the cell^23^. This approach was rebuilt (Fig. 9a) using aluminum foil instead of parafilm, as the contact between needle and parafilm could lead to contamination and as the needle showed strong bending during penetration of the elastic parafilm. Photographs of the setup can be found in Supplementary Fig. S1b. It was of interest, whether exposure of hydrophilic or hydrophobic samples to the high humidity atmosphere for several minutes affects the contact angle results. This possible influence was examined by measuring the SCA on one half of the sample, removing the droplets without contamination of the remaining sample area, storing the sample for 15 min in the water filled cell set up and measuring the contact angle afterwards on the other half. The experiment was repeated three times for each wetting state and Fig. 9b shows the results before and after exposure to the high humidity atmosphere. A hygrometer included in the chamber displayed a relative humidity of 75–80% at 22 °C in an additional experimental run.

The hydrophilic samples show a reaction to the high humidity atmosphere: After 15 min of storage in the glass cell the average SCA drops by 4% and is accompanied by a pronounced scattering of the single measurements. Shchedrina et al.^70^ argue that high humidity atmospheres may stop the organic carbon adsorption. Regarding the results in Fig. 9b this would not explain, why hydrophilic samples become more hydrophilic in the high humidity atmosphere. We believe that due to the hydrophilic character of the samples the surface reacts with the water molecules in the saturated atmosphere of the glass cell and a molecular water film forms on the surface. This creates a polar layer of water molecules resulting in a more hydrophilic behavior of the samples. Freund et al. showed^71^ that especially hydrophilic samples are prone to water film adsorption. They were only able to measure the thickness of the adsorbed film up to a relative humidity of 50% due to the meniscus forming. Extrapolation showed an expected film thickness of more than 140 nm at 80% relative humidity. The mechanism of water adsorption on metal surfaces is very complicated and influenced by different thermodynamic and kinetic parameters as the water does not form simple films consisting of water molecules on the surface, but might rather be prone to dissociation influenced by pre-absorbed oxygen^72–74^. On oxidized copper surfaces a molecular water adsorption could only be observed after a monolayered OH-film had formed^75^. In contrast, the hydrophobic samples do not seem to show any reaction with the water molecules in the surrounding atmosphere as the organic carbon contamination layer repels the water from the atmosphere and the sample keeps its hydrophobic SCA. For hydrophobic surfaces Freund et al.^71^ found out a reduced reaction with water below 50% relative humidity, but a slightly increased layer growth around 80% humidity of 50 nm with strong scattering. We believe that for the hydrophobic samples prolonged storage time in high humidity atmospheres would be necessary for the evolving thin water film to show any effect on the measured SCA. For low humidity around 2% barely any water film could be detected^71^ suggesting that the two week storage of the wrapped samples with silica gel beads suppressed the formation of a water layer and allowed the samples to adsorb the aliphatic carbon groups responsible for the hydrophobic behavior.

While for stable wetting states like fully aged hydrophobic samples exposure to high humidity atmospheres does not seem to influence the contact angle, metastable samples regarding wettability and surface composition might be influenced by a saturated atmosphere. An extended study focusing on the influence of different humidity levels on the adsorbate layer and therewith on the resulting contact angle would be necessary to quantify these effects.

The experiment conducted in Fig. 9 shows that the setup introduced by Drelich^23^ can be applied to hydrophobic samples but must be handled with care if the wetting behavior of the sample is still unknown or a hydrophilic behavior is expected.

#### Time of measurement

Once the droplet is applied on the surface under investigation, the change in contact angle with time must be considered. As mentioned in Ref.^22^, it can take some time for a droplet to reach a stable state on the surface but at the same time droplet evaporation is continuously decreasing the contact angle.

**Figure 10:**
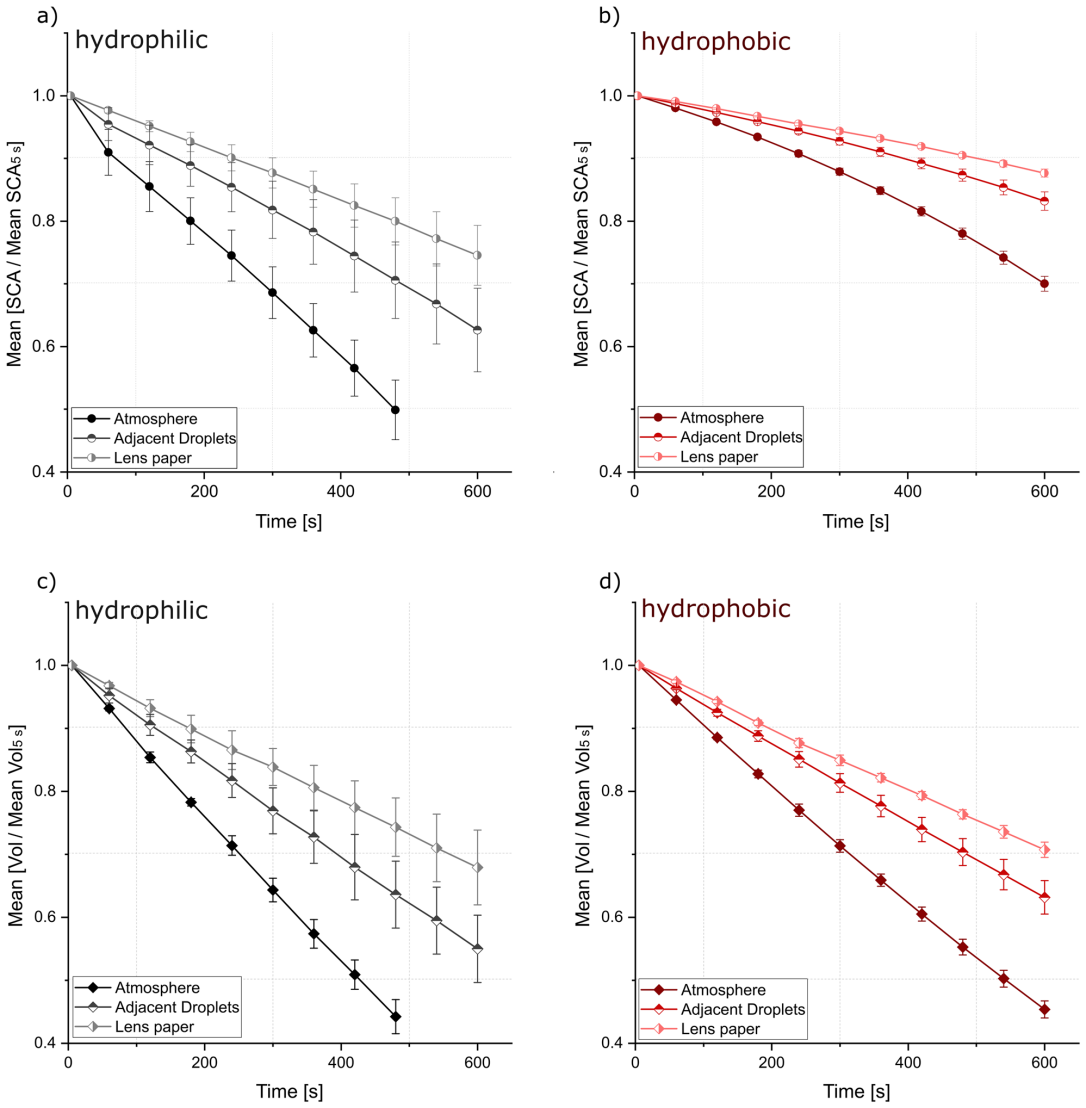
Change of the contact angle and volume for hydrophilic (**a,c**) and hydrophobic (**b,d**) copper surfaces through evaporation of 3 µl droplets under atmospheric conditions (Atmosphere), with adjacent droplets (Adjacent Droplets) and with wetted lens paper (Lens paper). Each experiment was repeated three times on different samples. Data for normalization is the contact angle/volume five seconds after droplet application. For better data visualization the normalized data for each of the three samples was averaged for every time step. Error bars show the standard deviation.

Figure 10 shows the development of the SCA and volume during evaporation of a 3 µl water droplet under ambient conditions (“atmosphere”), with increased surrounding humidity produced by droplets placed next to each other (“adjacent droplets”) and wet lens paper placed underneath the sample (“lens paper”). In general, the hydrophilic surface (Fig. 10a) shows a more pronounced reduction of the SCA under all conditions compared to the hydrophobic sample in Fig. 10b. In contrast, the reduction of droplet volume is similar for both sample types suggesting similar evaporation rates, independent of the initial wetting behavior of the surface. The hydrophilic samples seem to follow the constant-contact-radius evaporation mode introduced by Picknett and Bexon^76,77^ with a strong decrease of the SCA through evaporation. For the hydrophobic samples, the contact area between water and substrate might be subjected to a stronger reduction. For both sample wetting states the placement of adjacent droplets reduces the evaporation and therewith the reduction of the SCA and the volume effectively. A wet lens paper placed underneath the sample reduces the effect of evaporation even more. These two examples suggest that a high humidity micro-atmosphere created in immediate vicinity of the analyzed droplet can effectively reduce the negative effect of droplet evaporation on the measurement of SCAs without exposing the sample to the saturated atmosphere for several minutes before the measurement can take place.

**Figure 11:**
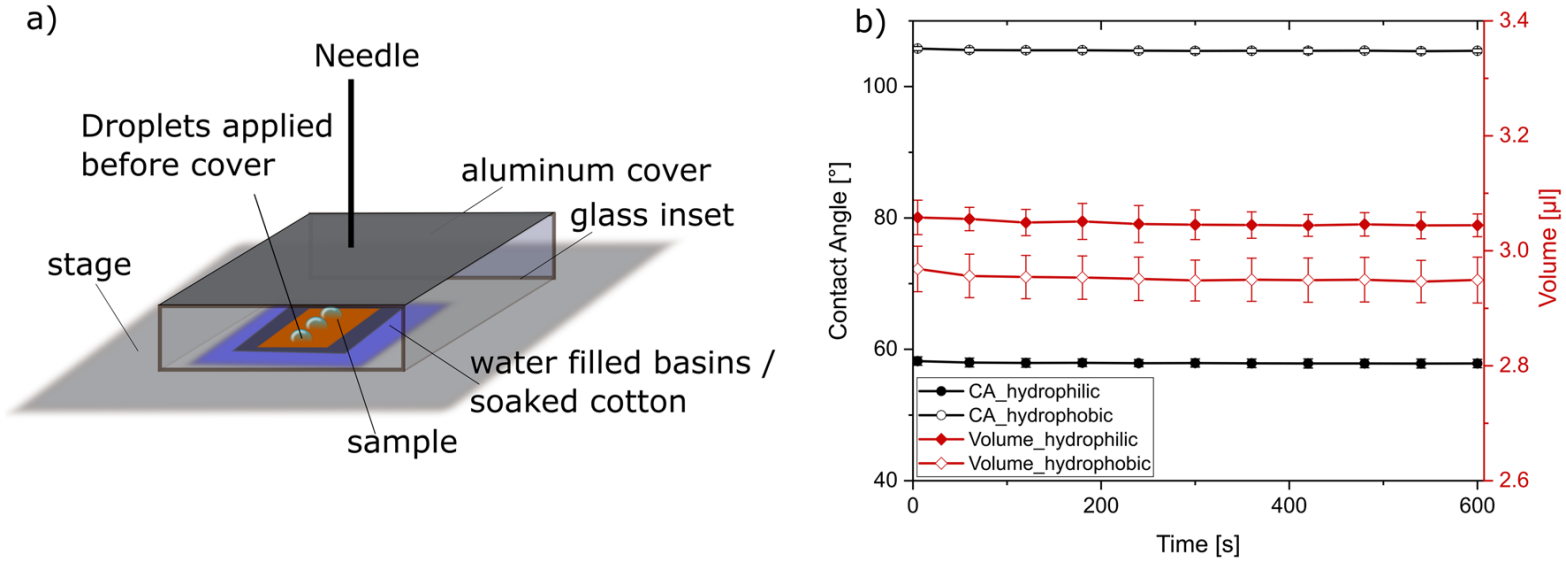
Schematic overview of the “cover”-setup (**a**). Change of the contact angle and the droplet volume, (**b**) over time with the designed setup (**a**). Each experiment was repeated three times on different samples.

Modern contact angle devices offer climate chambers to saturate the atmosphere and therewith avoid droplet evaporation. These chambers can be very expensive and especially for self-built contact angle devices hard to adapt. Moreover, as shown in Fig. 9, the exposure of surfaces to high humidity atmospheres before the measurement might alter the measured SCA depending on the initial wetting state of the sample. Therefore, we aimed at constructing a setup that allows for a suppression of droplet evaporation by creating a saturated micro-atmosphere around the droplet after droplet application. This is realized by a small cover fabricated out of glass or aluminum with glass inlets to allow for droplet acquisition after covering the sample. Additional sources of water under the cover should be provided either as small aluminum basins filled with water or by wet cotton placed next to the sample once all droplets are applied. A construction plan for a rotatable sample stage already equipped with integrated basins, together with a plan for an aluminum cover can be found in Supplementary Fig. S1c,d together with photographs of the setup (e and f). Schematics of the setup with experimental results to prove its success in the reduction of droplet evaporation are displayed in Fig. 11.

The reduction of the SCA after ten minutes is 0.3% for the hydrophobic samples and 0.6% for the hydrophilic samples. The volume is reduced by 0.7% and 0.5% respectively. These reductions are in the range of the standard deviation of the three measurement replicates and therewith negligible. The cover method displayed in Fig. 11 therewith successfully suppresses droplet evaporation as no relevant changes in the SCAs can be observed even after 10 min and allows for an observation of the SCA for several minutes, which can be especially important for patterned surfaces^11^.

**Figure 12:**
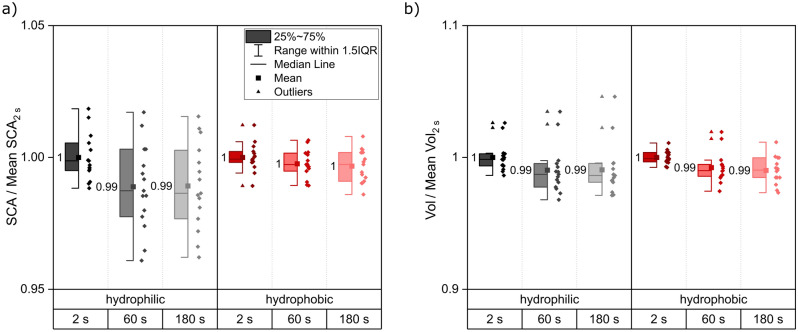
Static contact angle measurements with 3 µl of water. Five droplets per sample were analyzed on three samples each for both wetting states. Static contact angle (**a**) and volume (**b**) were measured 2 s, 60 s and 180 s after application. The reference data for normalization is “2 s”. For both wetting types a separate normalization was carried out. No significant differences were observed between the different measurement times suggesting stable measurement conditions.

For the experiments in Fig. 11 single droplets were applied on the surface and measured. In a detailed wetting analysis, multiple droplets usually are applied on the surface for statistical reasons and results are averaged. In that case for the “cover”-method all droplets must be applied before covering the sample, which should be done as quickly as possible to avoid evaporation. To assess the applicability of the setup under such real wetting analysis conditions, six droplets per sample were applied and measured right after droplet application and one minute and three minutes after droplet application. The first measurement 2 s after droplet application took place without the cover. Afterwards the sample was covered, and the remaining measurements (1 and 3 min) were conducted. The first droplet applied showed a reduction in droplet volume as it was exposed to the atmosphere without the cover for the longest time of all droplets. Therefore, the first droplet was not analyzed and served only as an adjacent droplet for the following ones which can further suppress evaporation as shown before in Fig. 10. Normalized results for the SCA and the volume are summarized in Fig. 12.

No relevant decrease in the SCA or the volume could be observed. The SCA of the hydrophilic samples decreases by only 1% while no change can be observed for the hydrophobic samples. On the hydrophilic samples, spreading is more likely to occur and could be a possible reason for the slight decrease of the SCA. The volume is reduced for both wetting types by 1%. The mentioned reductions are again below the typical standard deviation of multiple measurements. The conducted measurements prove that the newly presented “cover”-method is applicable in the wetting analyses for an observation of contact angles and droplet volume throughout several minutes. The “cover”-method aims at droplet application under ambient atmosphere, which is the relevant setup for any application. The cover design and combination with additional water sources next to the sample allows for a saturation of the atmosphere and therewith the observation of time dependent contact angle/droplet spreading behavior without exposure of the surface to high humidity before droplet application.

## Conclusions

This paper provides collected information on the possible influences on SCA measurements, which is the main method to analyze the wetting behavior of various materials and shows best practice examples as an approach towards reproducible SCA measurements in literature. Sample induced influences like preparation, cleaning and storage were discussed and experiments showed the extent of their influence on contact angle results for hydrophilic as well as hydrophobic samples. The two different wetting states were achieved by different storing conditions and times. Cellulose based wrapping paper proved to act as a carbon donor for the hydrophobization of copper surfaces.

For sample induced influences, a universal and objectively correct procedure is hard to be established. Therefore, it is in the responsibility of the researcher to provide defined sample states by controlling sample preparation, storage, and cleaning to ensure comparable conditions. In contrast, for the methodology of SCA measurements like applied droplet volume, application method, surrounding humidity and time of measurement clear experimental guidelines are necessary to ensure comparability of different studies. The quality of the water used for the measurement only showed an effect on the hydrophilic samples, where a stronger interaction with the substrate is assumed in contrast to the hydrophobic samples with a more pronounced protection layer of adventitious carbon. Increasing the droplet volume from 3 to 6 µl already decreases the contact angle due to gravity effects. We recommend dosing 3 µl of purified water as a standard measure between larger volumes that are influenced by gravity and lower volumes that might be heavily distorted by the needle^22^. However, sample surface condition must be considered especially for topographically or chemically heterogeneous surfaces as the droplet should be large enough to equal out local inhomogeneities^25^. It was further shown that droplet application can either be conducted by the needle approaching the sample until the droplet wets the surface or by the stage picking up the droplet. Pipetting of droplets should only be performed, if needle and stage application are not possible as the enhanced pressure applied by the researcher can lead to droplet spreading and smaller SCAs especially on hydrophilic samples. The presented results show that evaporation affects the contact angle results drastically within a few minutes. Hence it is important to perform measurements as quickly as possible after droplet application if no measures are taken to suppress evaporation. Still, a few seconds are needed to balance out vibrations from droplet applications - 5 s proved useful here. If evaporation is to be reduced e.g., for the time dependent observation of the droplet, placing a wet lens paper underneath the sample can be helpful. If a longer observation of the droplet is desired to, for example examine spreading behavior or to allow for the droplet to reach a stable wetting state, evaporation should be controlled. Exposing samples to a saturated atmosphere before droplet application as suggested by Drelich^23^ proved to potentially influence the contact angle for hydrophilic samples. Aged hydrophobic samples did not show a visible reaction to the high humidity. In general, the conducted experiments showed that hydrophobic samples seem most robust against environmental influences. It is believed that this is due to the adventitious carbon layer covering the substrate and acting as a protection against environmental influences. To avoid any possible influence of droplet evaporation or high humidity exposure, this paper demonstrates a new measurement setup allowing for droplet application under atmospheric conditions often equal to sample storage conditions and therewith offers the chance to correlate results to other measurements like XPS. Covering the applied droplets after application in the shown setup appears reliable in terms of reproducible contact angle measurements. This study not only discusses possible influences on the analyses of wetting properties, but also experimentally proves the extent of the effects and gives clear advice for setting the measurement parameters. The newly introduced “cover”-method allows for a droplet application under atmospheric conditions and an observation of the wetting behavior for several minutes by successfully suppressing droplet evaporation.

## Supplementary Information

Supplementary Information.

## Data Availability

Data is available upon reasonable request from the corresponding author.

## References

[1] Bryk P (2020). What is the value of water contact angle on silicon?. Materials.

[2] Korczeniewski E (2021). Revisiting wetting, freezing, and evaporation mechanisms of water on copper. ACS Appl. Mater. Interfaces.

[3] Li G (2008). Stable superhydrophobic surface: Fabrication of interstitial cottonlike structure of copper nanocrystals by magnetron sputtering. Sci. Technol. Adv. Mater..

[4] Brennan WJ, Feast WJ, Munro HS, Walker SA (1991). Investigation of the ageing of plasma oxidized PEEK. Polymer.

[5] Wagner N, Theato P (2014). Light-induced wettability changes on polymer surfaces. Polymer.

[6] Aria AI (2016). Time evolution of the wettability of supported graphene under ambient air exposure. J. Phys. Chem. C.

[7] Rafiee J (2012). Wetting transparency of graphene. Nat. Mater..

[8] Ranc H (2006). Friction coefficient and wettability of oral mucosal tissue: Changes induced by a salivary layer. Colloids Surf. A.

[9] Marmur A, Della Volpe C, Siboni S, Amirfazli A, Drelich JW (2017). Contact angles and wettability: Towards common and accurate terminology. Surf. Innov..

[10] Kam DH, Bhattacharya S, Mazumder J (2012). Control of the wetting properties of an AISI 316L stainless steel surface by femtosecond laser-induced surface modification. J. Micromech. Microeng..

[11] Lößlein SM, Mücklich F, Grützmacher PG (2021). Topography versus chemistry—How can we control surface wetting?. J. Colloid Interface Sci..

[12] Hans M, Müller F, Grandthyll S, Hüfner S, Mücklich F (2012). Anisotropic wetting of copper alloys induced by one-step laser micro-patterning. Appl. Surf. Sci..

[13] Müller DW (2020). In-depth investigation of copper surface chemistry modification by ultrashort pulsed direct laser interference patterning. Langmuir.

[14] Xia D, Johnson LM, López GP (2012). Anisotropic wetting surfaces with one-dimesional and directional structures: Fabrication approaches, wetting properties and potential applications. Adv. Mater..

[15] Young T (1805). An essay on the cohesion of fluids. Philos. Trans. R. Soc..

[16] Wenzel RN (1936). Resistance of solid surfaces to wetting by water. Ind. Eng. Chem..

[17] Cassie ABD, Baxter S (1944). Wettability of porous surfaces. Trans. Faraday Soc..

[18] Horsthemke A, Schröder JJ (1985). The wettability of industrial surfaces: Contact angle measurements and thermodynamic analysis. Chem. Eng. Process..

[19] Heier M (2021). Experimental study of the influence of the adsorbate layer composition on the wetting of different substrates with water. Adsorpt. Sci. Technol..

[20] Long J, Zhong M, Fan P, Gong D, Zhang H (2015). Wettability conversion of ultrafast laser structured copper surface. J. Laser Appl..

[21] Rudawska A, Jacniacka E (2009). Analysis for determining surface free energy uncertainty by the Owen-Wendt method. Int. J. Adhes. Adhes..

[22] Huhtamäki T, Tian X, Korhonen JT, Ras RHA (2018). Surface-wetting characterization using contact-angle measurements. Nat. Protoc..

[23] Drelich J (2013). Guidelines to measurements of reproducible contact angles using a sessile-drop technique. Surf. Innov..

[24] Eral HB, t Mannetje DJCM, Oh JM (2013). Contact angle hysteresis: A review of fundamentals and applications. Colloid Polym. Sci..

[25] Marmur A (2009). Solid-surface characterization by wetting. Annu. Rev. Mater. Res..

[26] Kung CH, Sow PK, Zahiri B, Mérida W (2019). Assessment and interpretation of surface wettability based on sessile droplet contact angle measurement: Challenges and opportunities. Adv. Mater. Interfaces.

[27] Müller DW (2021). Increasing antibacterial efficiency of cu surfaces by targeted surface functionalization via ultrashort pulsed direct laser interference patterning. Adv. Mater. Interfaces.

[28] Davis JR, Committee ASMIH (2001). Copper and Copper Alloys.

[29] Vincent M (2016). Antimicrobial applications of copper. Int. J. Hyg. Environ. Health.

[30] Cheng Y (2018). Controllable fabrication of superhydrophobic alloys surface on copper substrate for self-cleaning, anti-icing, anti-corrosion and anti-wear performance. Surf. Coat. Technol..

[31] Lößlein SM (2021). Patience alone is not enough—A guide for the preparation of low-defect sections from pure copper. Pract. Metallogr..

[32] Krüss. *Kegelschnittmethode*. https://www.kruss-scientific.com/de-DE/know-how/glossar/kegelschnittmethode. Accessed 29 August 2022.

[33] Baumann R (2021). Tailored wetting of copper using precise nanosecond direct laser interference patterning. Opt. Lasers Eng..

[34] Milles S, Voisiat B, Nitschke M, Lasagni AF (2019). Influence of roughness achieved by periodic structures on the wettability of aluminum using direct laser writing and direct laser interference patterning technology. J. Mater. Process. Technol..

[35] Rosenkranz A (2015). Oxide formation, morphology, and nanohardness of laser-patterned steel surfaces: Oxide formation, morphology, and nanohardness. Adv. Eng. Mater..

[36] Kietzig A-M, NegarMirvakili M, Kamal S, Englezos P, Hatzikiriakos SG (2011). Laser-patterned super-hydrophobic pure metallic substrates: Cassie to Wenzel wetting transitions. J. Adhes. Sci. Technol..

[37] Allahyari E (2019). Laser surface texturing of copper and variation of the wetting response with the laser pulse fluence. Appl. Surf. Sci..

[38] Milles S (2021). Wetting properties of aluminium surface structures fabricated using direct laser interference patterning with picosecond and femtosecond pulses. JLMN..

[39] Pazokian H (2012). Tailoring the wetting properties of polymers from highly hydrophilic to superhydrophobic using UV laser pulses. J. Micromech. Microeng..

[40] Waugh D, Lawrence J, Langer N, Bidault S (2018). Mixed-state wetting and wetting transitions on laser surface engineered polymeric materials. Int. J. Wettabil. Sci. Technol..

[41] Murakami D, Jinnai H, Takahara A (2014). Wetting transition from the Cassie-Baxter state to the Wenzel state on textured polymer surfaces. Langmuir.

[42] Grewal HS, Cho I-J, Oh J-E, Yoon E-S (2014). Effect of topography on the wetting of nanoscale patterns: Experimental and modeling studies. Nanoscale.

[43] Shirazy MRS, Blais S, Fréchette LG (2012). Mechanism of wettability transition in copper metal foams: From superhydrophilic to hydrophobic. Appl. Surf. Sci..

[44] Yang Z, Liu X, Tian Y (2019). Insights into the wettability transition of nanosecond laser ablated surface under ambient air exposure. J. Colloid Interface Sci..

[45] Beamson G, Briggs D (1992). High Resolution XPS of Organic Polymers the Scienta ESCA300 Database.

[46] Platzman I, Brener R, Haick H, Tannenbaum R (2008). Oxidation of polycrystalline copper thin films at ambient conditions. J. Phys. Chem. C.

[47] Preston DJ (2014). Effect of hydrocarbon adsorption on the wettability of rare earth oxide ceramics. Appl. Phys. Lett..

[48] Kim D, Kim JG, Chu CN (2016). Aging effect on the wettability of stainless steel. Mater. Lett..

[49] Chang F-M, Cheng S-L, Hong S-J, Sheng Y-J, Tsao H-K (2010). Superhydrophilicity to superhydrophobicity transition of CuO nanowire films. Appl. Phys. Lett..

[50] ASTM D1193–06(2018) (2018). Standard specification for reagent water. Book Stand..

[51] Goel S (2019). Water and Wastewater Engineering.

[52] Bonn D, Eggers J, Indekeu J, Meunier J, Rolley E (2009). Wetting and spreading. Rev. Mod. Phys..

[53] Voué M, De Coninck J (2000). Spreading and wetting at the microscopic scale: Recent developments and perspectives. Acta Mater..

[54] Bizi-Bandoki P, Benayoun S, Valette S, Beaugiraud B, Audouard E (2011). Modifications of roughness and wettability properties of metals induced by femtosecond laser treatment. Appl. Surf. Sci..

[55] Pu Z, Jing X, Yang C, Wang F, Ehmann KF (2020). Wettability modification of zirconia by laser surface texturing and silanization. Int. J. Appl. Ceram. Technol..

[56] Elleb R (2021). Study of femtosecond laser multi-scale textured steel surfaces on the wettability in relation to ageing. J. Mater. Sci..

[57] Zhang J (2021). Modified wettability of micro-structured steel surfaces fabricated by elliptical vibration diamond cutting. Int. J. Precis. Eng. Manuf.-Green Technol..

[58] Fu Y (2020). Wettability control of polymeric microstructures replicated from laser-patterned stamps. Sci. Rep..

[59] Kuznetsov GV (2021). Influence of roughness on polar and dispersed components of surface free energy and wettability properties of copper and steel surfaces. Surf. Coat. Technol..

[60] Altgen D, Altgen M, Kyyrö S, Rautkari L, Mai C (2020). Time-dependent wettability changes on plasma-treated surfaces of unmodified and thermally modified European beech wood. Eur. J. Wood Prod..

[61] Seo K, Kim M, Ahn JK, Kim DH (2015). Effects of drop size and measuring condition on static contact angle measurement on a superhydrophobic surface with goniometric technique. Korean J. Chem. Eng..

[62] Zhang D (2012). A simple way to achieve pattern-dependent tunable adhesion in superhydrophobic surfaces by a femtosecond laser. ACS Appl. Mater. Interfaces.

[63] Drelich J, Miller JD, Hupka J (1993). The effect of drop size on contact angle over a wide range of drop volumes. J. Colloid Interface Sci..

[64] Letellier P, Mayaffre A, Turmine M (2007). Drop size effect on contact angle explained by nonextensive thermodynamics. Young’s equation revisited. J. Colloid Interface Sci..

[65] Chen Y, He B, Lee J, Patankar NA (2005). Anisotropy in the wetting of rough surfaces. J. Colloid Interface Sci..

[66] May A (2015). Laser induced anisotropic wetting on Ti–6Al–4V surfaces. Mater. Lett..

[67] Rosenkranz A (2016). Synergetic effect of laser patterning and micro coining for controlled lubricant propagation. Surf. Topogr. Metrol. Prop..

[68] Wu H (2019). Large area metal micro-/nano-groove arrays with both structural color and anisotropic wetting fabricated by one-step focused laser interference lithography. Nanoscale.

[69] Zhao Q, Liu Y, Abel EW (2004). Effect of temperature on the surface free energy of amorphous carbon films. J. Colloid Interface Sci..

[70] Shchedrina N (2020). Wetting angle stability of steel surface structures after laser treatment. Opt. Quant. Electron..

[71] Freund J, Halbritter J, Hörber JK (1999). How dry are dried samples? Water adsorption measured by STM. Microsc Res Tech.

[72] Hodgson A, Haq S (2009). Water adsorption and the wetting of metal surfaces. Surf. Sci. Rep..

[73] Spitzer A, Lüth H (1985). An XPS study of the water adsorption on Cu(110). Surf. Sci..

[74] Spitzer A, Lüth H (1982). The adsorption of water on clean and oxygen covered Cu(110). Surf. Sci..

[75] Trotochaud L (2018). Water adsorption and dissociation on polycrystalline copper oxides: Effects of environmental contamination and experimental protocol. J. Phys. Chem. B.

[76] Picknett RG, Bexon R (1977). The evaporation of sessile or pendant drops in still air. J. Colloid Interface Sci..

[77] Hasan MS, Sobolev K, Nosonovsky M (2021). Evaporation of droplets capable of bearing viruses airborne and on hydrophobic surfaces. J. Appl. Phys..

