# Supplementary material for: An in-depth evaluation of sample and measurement induced influences on static contact angle measurements
Source: Sci Rep. 2022 Nov 12;12:19389. doi: 10.1038/s41598-022-23341-3 (PMC9653445; doi:10.1038/s41598-022-23341-3)
Supplement: Supplementary file 1 — Supplementary Information. [file 41598_2022_23341_MOESM1_ESM.pdf]

Supplementary Material

An in-depth Evaluation of Sample and Measurement induced Influences on Static Contact Angle Measurements

Sarah Lößlein[1,*], Rolf Merz[2], Daniel Müller[1], Michael Kopnarski[2], Frank Mücklich[1]

1 Chair of Functional Materials, Department of Material Science and Engineering, Campus D3 3, 66123 Saarbrücken, Germany

2 Institute for Surface and Thin Film Analysis, Technische Universität Kaiserslautern, Germany

Correspondence: sarah.loesslein@uni-saarland.de

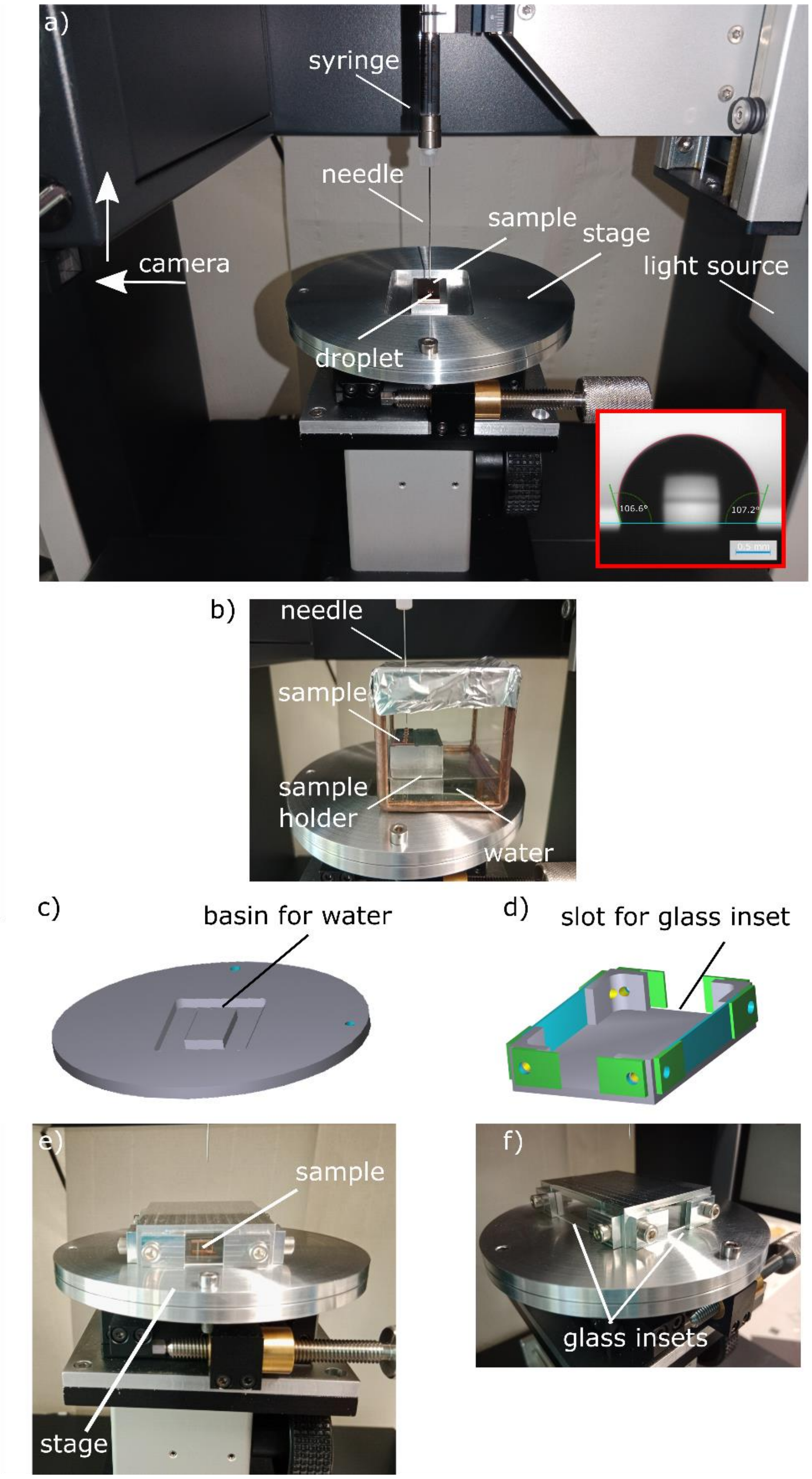

Supplementary Figure S1: (a) Photograph of the experimental setup used for static contact angle measurements on a *Krüss DSA 100*. The inset shows a recorded image of a droplet applied on a hydrophobic copper surface with an ellipse fit in the *Krüss Advance software*. (b) Photograph of the experimental setup suggested by Drelich[1] to suppress droplet evaporation. (c) CAD-file of an aluminum stage for static contact angle measurements with milled basins for water. (d) CAD-file of an aluminum cover with slots for glass insets. Sizes should scale with sample size keeping the cover height and width as small as possible and the basins as big as possible. (e, f) Photographs of the stage filled with water and droplets placed on a copper sample covered with the designed aluminum cover.

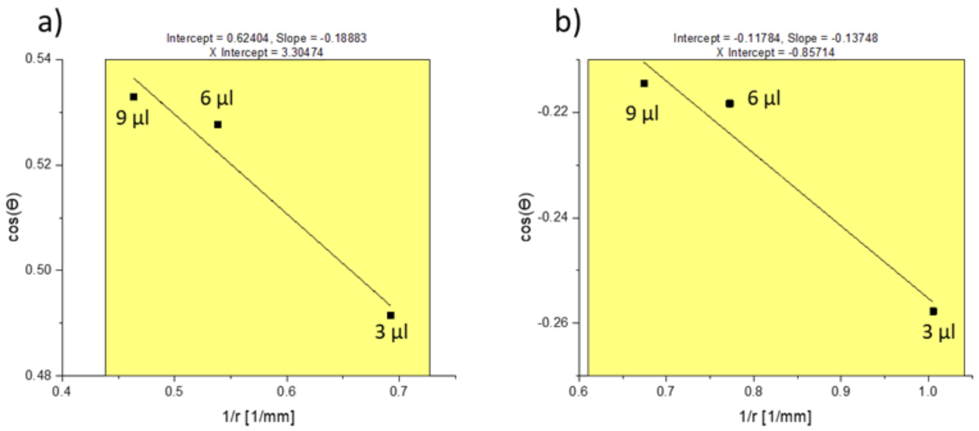

Supplementary Figure S2: Cosine of the averaged SCA for hydrophilic (a) and hydrophobic (b) samples over the inverse droplet radius as a measure for line tension. For both wetting types a linear correlation can be assumed supporting the thesis of a constant line tension and therewith independence of the shown droplet volumes [2].

Supplementary Table S1: Example for calculation of sample wise data normalization for three samples (α, β, γ) with three applied droplets each. Three images were taken from each droplet averaging to the SCA of the droplets. The mean of these individual SCAs on the reference samples delivers the *Mean* $SCA_{Reference}$ (per sample). Whenever possible, on the same sample, the parameter under investigation was applied and new droplets were measured (SCA). The normalized data was calculated by dividing SCA by *Mean* $SCA_{Reference}$ of the respective sample.

Sample	Reference				Measurement				Normalized Data
	Single image SCA			SCA	Single image SCA			SCA	
α	a_1	a_2	a_3	Mean [a_1;a_2;a_3] = a	A_1	A_2	A_3	Mean [A_1;A_2;A_3] = A	A/x
	b_1	b_2	b_3	Mean [b_1;b_2;b_3] = b	B_1	B_2	B_3	Mean [B_1;B_2;B_3] = B	B/x
	c_1	c_2	c_3	Mean [c_1;c_2;c_3] = c	C_1	C_2	C_3	Mean [C_1;C_2;C_3] = C	C/x
	Mean $SCA_{Reference}$ = Mean [a;b;c] = x								
β	d_1	d_2	d_3	Mean [d_1;d_2;d_3] = d	D_1	D_2	D_3	Mean [D_1;D_2;D_3] = D	D/y
	e_1	e_2	e_3	Mean [e_1;e_2;e_3] = e	E_1	E_2	E_3	Mean [E_1;E_2;E_3] = E	E/y
	f_1	f_2	f_3	Mean [f_1;f_2;f_3] = f	F_1	F_2	F_3	Mean [F_1;F_2;F_3] = F	F/y
	Mean $SCA_{Reference}$ = Mean [d;e;f] = y								
γ	g_1	g_2	g_3	Mean [g_1;g_2;g_3] = g	G_1	G_2	G_3	Mean [G_1;G_2;G_3] = G	G/z
	h_1	h_2	h_3	Mean [h_1;h_2;h_3] = h	H_1	H_2	H_3	Mean [H_1;H_2;H_3] = H	H/z
	i_1	i_2	i_3	Mean [i_1;i_2;i_3] = i	I_1	I_2	I_3	Mean [I_1;I_2;I_3] = I	I/z
	Mean $SCA_{Reference}$ = Mean [g;h;i] = z								

References

1. Drelich, J. Guidelines to measurements of reproducible contact angles using a sessile-drop technique. *Surface Innovations* **1**, 248–254 (2013).
2. Drelich, J., Miller, J. D. & Hupka, J. The Effect of Drop Size on Contact Angle over a Wide Range of Drop Volumes. *Journal of Colloid and Interface Science* **155**, 379–385 (1993).
